# Valuing the Endangered Species *Antirrhinum lopesianum*: Neuroprotective Activities and Strategies for *in vitro* Plant Propagation

**DOI:** 10.3390/antiox2040273

**Published:** 2013-10-28

**Authors:** Andreia Gomes, Sofia Fortalezas, Rui Pimpão, Inês Figueira, João Maroco, Carlos Aguiar, Ricardo B. Ferreira, Célia Miguel, Cláudia N. Santos

**Affiliations:** 1 Institute of Chemical and Biological Technology, New University of Lisbon, Av. da República, Oeiras 2780-157, Portugal; E-Mails: andreiagomes@itqb.unl.pt (A.G.); fortalezas@itqb.unl.pt (S.F.); pimpaorc@itqb.unl.pt (R.P.); inesf@itqb.unl.pt (I.F.); rbferreira@itqb.unl.pt (R.B.F.); cmiguel@itqb.unl.pt (C.M.); 2 Institute of Experimental and Technological Biology, Apartado 12, Oeiras 2781-901, Portugal; 3 University Institute of Psychological, Social and Life Sciences, Rua Jardim do Tabaco 34, Lisboa 1149-051, Portugal; E-Mail: joao.maroco@ispa.pt; 4 Mountain Research Center, Polytechnic Institute of Bragança, Apartado 1172, Bragança 5301-855, Portugal; E-Mail: cfaguiar@ipb.pt; 5 Department of Botany and Biological Engineering, Institute of Agronomy, Technical University of Lisbon, Tapada da Ajuda, Lisboa 1349-017, Portugal

**Keywords:** *Antirrhinum lopesianum*, phytochemical profile, antioxidant capacity, neuroprotection, acetylcholinesterase inhibitor, *ex situ* conservation

## Abstract

Plant phytochemicals are described as possessing considerable neuroprotective properties, due to radical scavenging capacity and acetylcholinesterase inhibitory activity, important bioactivities in neurodegeneration. *Antirrhinum lopesianum* is a rare endemism from the Iberian Peninsula, occurring at the northeastern border between Portugal and Spain. It is classified as Endangered, due to its highly fragmented geographical occupation, facing a high risk of extinction in the Portuguese territory, within 20 years. Here, we describe for the first time the chemical characterization of extracts of the species concerning total phenol content, flavonoid content and antioxidant properties. The profile of high performance liquid chromatography with diode array detector (HPLC-DAD) of the polyphenol-enriched fraction of plant extracts was also performed, showing the great potential of the species as a source of bioactive phytochemical compounds. *A*. *lopesianum*’s potential for neuroprotection was revealed by a significant acetylcholinesterase inhibitory activity and also by a neuroprotective effect on a human cell model of neurodegeneration. Moreover, this is the first report describing a successful procedure for the *in vitro* propagation of this endangered species. The comparison of phenolic content and the HPLC-DAD profile of wild and *in vitro* propagated plants revealed that *in vitro* plants maintain the ability to produce secondary metabolites, but the profiles are differentially affected by the growth regulators. The results presented here greatly contribute to the value for this species regarding its potential as a source of phytochemicals with prospective neuroprotective health benefits.

## 1. Introduction

Plants are important sources of new drug molecules. About 49% of new drugs, developed between 1981 and 2002, were either natural products or derived from them as semi-synthetic derivatives. Plant secondary metabolites have long contributed to the development of small-molecule therapeutics, due, in part, to their combination of unique chemical features and potent bioactivities, from antioxidant to anti-cancer compounds [1]. Only about 10% of the existing higher plant species have been chemically characterized, so the unknown diversity of the plant kingdom represents an immense reservoir of molecules with potential pharmacological value [2]. Importantly, endangered plant species not yet characterized may hide beneficial health effects to be discovered, it being vital to characterize them before losing, and, most importantly, also to preserve, them [3,4].

One main focus area where plant secondary metabolite bioactivities have been shown to be effective is for the treatment of degenerative diseases [5]. Several small-molecules have been reported to exhibit inhibitory properties in neurodegeneration [6,7,8].

Neurodegenerative disorders have an enormous economic and social costs [9,10]. The predicted increase in its incidence, due to an ageing population, together with the lack of any cure, make the development of new therapies for halting or reversing such diseases urgent, plants extracts being very attractive options. These diseases are multifactorial disorders in which many biological processes become unregulated. Therefore, a multi-target therapeutic strategy aiming at different pharmacological mechanisms might provide a more rational and improved dementia treatment approach.

Alterations produced by neuropathologies include oxidative stress markers [11], as well as the decline in cognitive function associated with cholinergic deficits [12]. The limited resources for combating oxidative stress by the central nervous system include: vitamins, bioactive molecules, lipoic acid, antioxidant enzymes and redox sensitive protein transcriptional factors. Furthermore, this defense system can be activated/modulated by natural products, such as polyphenols [6,7]. Moreover acetylcholinesterase (AChE) inhibition may help in the treatment of Alzheimer’s disease (AD), as well as senile dementia, myasthenia gravis [13], Parkinson’s disease [14] and ataxia [15], due to the associated cholinergic deficit [12]. Few inhibitors have yet been approved for AD therapy [12], and most of them have a short half-life and peripheral cholinergic side-effects [15,16,17], which significantly limit its therapeutic use. Therefore, natural sources of compounds showing AChE inhibitory activity must be sought.

*Antirrhinum lopesianum* is a plant listed as endangered, since it presents a highly fragmented distribution, with a low area of occupancy, below 500 km^2^ [18]. A probability of extinction of 33% within 20 years has been predicted for the species in its largest subpopulation, and prospects for the other subpopulations are probably worse [19,20].

*A*. *lopesianum* was collected for the first time in 1877 or 1879 by Manuel Ferreira, a plant collector of the Coimbra University, nearby the city of Bragança (northeast of Portugal), and it was identified as *A*. *molle* [21]. The amateur botanist Priest Miranda Lopes found it again in 1926 in the valley of the Maçãs river [22], about twenty kilometers away, towards the SW, from the population originally discovered by M. Ferreira. In his monograph of the genus *Antirrhinum* of 1956, the German botanist Werner Rothmaler recognized the taxonomic autonomy of this *Antirrhinum* and dedicated the new species—*Antirrhinum lopesianum* Rothm.—to Priest Miranda Lopes [23]. *A*. *lopesianum* was not collected again throughout the following seventy years, and the exact localization of the once collected populations was uncertain. It was rediscovered, and new populations recorded, in 1990 [24]. Amich *et. al*. (1989) [20] located the first Spanish population in the Douro River International Canyon.

*A*. *lopesianum* is presently narrowly endemic to the NW of the Iberian Peninsula, specifically, the hydrographic basin of the Sabor River and the international stretch of the Douro River valley [18,25]. This plant is a perennial chamaephyte that inhabits shaded cracks and is a calcicolous, endemic to a territory totally dominated by acid rocks [24]. The species is severely fragmented in ten known populations with a total area of occupancy of about 29,000 m^2^ (13,000 and 16,000 m^2^ in Portugal and Spain, respectively) [25]. *A*. *lopesianum* has been listed as a threatened species in the Habitats Directive 92/43/EEC [26]. Its conservation status based on International Union for Conservation of Nature (IUCN) [19] was evaluated as critically endangered in Portugal and as endangered in Spain [25]. Moreover, the recently published European Red List of Vascular Plants attributed to *A*. *lopesianum* the status of endangered [27].

*In vitro* propagation of endangered plants can represent an attractive strategy for *ex situ* conservation, ensuring rapid cultivation of species that have become vulnerable for various reasons, including the degradation of their natural habitats or their limited reproductive capacity. Techniques for *in vitro* vegetative propagation and preservation are essential components of plant genetic resource management, and they are becoming increasingly important for the conservation of rare and endangered plants [28,29,30,31,32,33].

In the present work, a combined approach was followed to characterize the species in terms of chemical composition and to devise strategies for *ex situ* preservation, relying on seed conservation and *in vitro* culture. The phytochemical characterization and the identification of compounds with potential bioactivity will contribute to the recovery of this species, ultimately contributing to its conservation. An efficient system for *in vitro* vegetative propagation, as well as for acclimatization has been established, followed by a pilot experiment of reintroduction into the natural habitat. Furthermore, the assessment of the potential of *A*. *lopesianum* as a source of phenolics and flavonoids has been performed with plant extracts prepared from plants growing under natural conditions and from *in vitro* plants, in order to evaluate if the plant properties are preserved after *in vitro* culture. Evaluation of the neuroprotective potential of *A*. *lopesianum* extracts through the use of a neurodegeneration cell model and the assessment of acetylcholinesterase (AChE) inhibitory activity contributes to the value of the species as a natural source of neuroprotective compounds.

## 2. Experimental Section

### 2.1. Plant Material

*Antirrhinum lopesianum* life specimens and seeds were collected from their wild habitat, in Bragança region (Alfaião, Bragança, Portugal, altitude 502 m; latitude: 41.75904; longitude: −6.70401. May 13, 2011. C. Aguiar without No. BRESA—herbarium of Escola Superior Agrária de Bragança—7405), at a full mature stage. Samples of plant material, namely leaves and stems, were mashed together and freeze-dried until analysis.

Plants were collected for two consecutive years for total phenolic content, total flavonoid and antioxidant capacity. Second year plants were collected for acetylcholinesterase activity, cytotoxicity and neuroprotection. The HPLC profile was done for second year plants and *in vitro* propagated plants.

In the case of plant material derived from *in vitro* propagated specimens of *A*. *lopesianum*, fully expanded leaves and stems were collected immediately after acclimatization. Both organs were mashed together and freeze-dried until analysis.

### 2.2. Chemical Characterization

#### 2.2.1. Extraction of Plant Phytomolecules

The extraction of plant phytomolecules was performed as described earlier [34,35]. Briefly, to each 1 g of lyophilized powder, 12 mL of hydroethanolic solvent (50% (v/v) ethanol/water) was added, and the mixture was shaken for 30 min at room temperature in the dark. The mixture was then centrifuged at 12,400× *g* for 10 min at room temperature. The supernatant was filtered through paper filter and then through 0.2 µm cellulose acetate membrane filters. The resulting extracts were stored frozen at −80 °C.

#### 2.2.2. Fractionation by Solid Phase Extraction

Hydroethanolic extracts were fractionated by solid phase extraction (SPE) using a Giga tubes 2 g/12 mL, C18-E unit (Phenomenex^®^), as described before [35]. Briefly, columns were pre-washed in 0.5% (v/v) glacial acetic acid in acetonitrile and then pre-equilibrated in 0.5% (v/v) glacial acetic acid in water. The extracts were dried under vacuum and resuspended in 0.5% (v/v) glacial acetic acid in water; then, they were applied to the columns and unbound material, which contained the free sugars, organic acids and minerals, and was discarded. The columns were washed with 0.5% (v/v) aqueous acetic acid, and then, polyphenol-enriched bound fractions were eluted with 0.5% (v/v) glacial acetic acid in acetonitrile.

#### 2.2.3. Total Phenolic Content

Determination of total phenolic compounds was performed by the Folin-Ciocalteu method adapted to a microplate reader, as described in previous work [35,36]. Gallic acid was used as the standard, and results are expressed in milligrams of gallic acid equivalents per gram of dry weight (mg GAE g^−1^ dw) of plant material.

#### 2.2.4. Total Flavonoid Content

Measurement of total flavonoid content was performed by a modification of the AlCl_3_ complexation method, as described before [35].

#### 2.2.5. HPLC Profile

HPLC analysis of *A*. *lopesianum* hydroethanolic extracts, followed by SPE fractionation, were conducted on a Hitachi HPLC instrument (VWR) equipped with EZChrom Elite software (Agilent), a model L-2130 pump system, a model L-2200 autosampler, a model L2300 column oven and a model L2455 DAD system. A sample volume of 10 µL was injected, and separations were achieved on an Inertsil ODS-3V column (250 × 4.6 mm, 5 µm), operated at 30 °C. The mobile phase consisted in 0.1% (v/v) formic acid in water (solvent A) and acetonitrile (solvent B). A flow rate of 1 mL min^−1^ was used. The gradient started with 5% (v/v) B to reach 40% (v/v) B at 60 min and 100% (v/v) B at 66 min, where it stayed until 76 min. Chromatograms were recorded at 280, 320, 370 and 520 nm from diode array data collected between 200 and 600 nm. The UV absorbance spectrum of peaks was used for characterization and identification of compound groups by comparison with the literature.

### 2.3. Bioactivities Assessment

#### 2.3.1. Antioxidant Capacity

The antioxidant capacity of plant extracts was determined by the Oxygen Radical Absorbance Capacity method (ORAC) adapted to a microplate, as described earlier [35,36]. Trolox was used as the standard, and results are expressed in micromoles of trolox equivalents per gram of plant dry weight (μmol TE g^−1^ dw).

#### 2.3.2. Acetylcholinesterase (AChE) Inhibitory Assay

AChE inhibition of *A*. *lopesianum* hydroethanolic extract and polyphenol-enriched fraction (PEF) was determined in a 96-microtiter well plate, based on Ellman’s reaction [37], according to [38]. The effect on AChE activity was calculated as an inhibition percentage (%) of the maximum activity (registered on control wells without the inhibitor).

#### 2.3.3. Cytotoxicity Profile

*A*. *lopesianum* PEF was concentrated under vacuum and dissolved in SK-N-MC cell medium for the cytotoxicity tests [35,39]. The cell viability assay was performed in a 96-well plate cell and employed the neuroblastoma human cell line SK-N-MC to identify the non-toxic range of extract concentrations. Cells were seeded at 1.25 × 10^5^ cells mL^−1^ and grown for 24 h prior to incubation with extracts. Toxicity tests involved 24 h incubation in the range 0–200 µg GAE mL^−1^ of medium. Cell viability was assessed using the CellTiter-Blue^®^ Cell Viability Assay (Promega), according to the manufacturer’s instructions. Non-viable cells rapidly lose their metabolic capacity and, thus, do not generate the fluorescent signal.

#### 2.3.4. Neuroprotective Effect

To evaluate the neuroprotective effect of extracts, SK-N-MC neuroblastoma cells were incubated in the presence of H_2_O_2_ [39]. Briefly, cells were seeded at 7.4 × 10^4^ cells mL^−1^, and 24 h after seeding, the growth medium was removed and the wells washed with phosphate buffered saline (PBS). Cells were pre-incubated with medium containing 0.5% (v/v) fetal bovine serum (FBS) supplemented with non-toxic concentrations of *A*. *lopesianum* PEF. After 24 h of pre-incubation, cells were washed again with PBS, and the medium was replaced by medium containing 0.5% (v/v) FBS and H_2_O_2_ at a final concentration of 300 µM. After 24 h, neuroprotective potential viability was assessed using the CellTiter-Blue^®^ Cell Viability Assay (Promega).

### 2.4. *In vitro* Establishment and Propagation

#### 2.4.1. Seed Disinfection and Germination

Collected seeds were surface-sterilized by immersion in a 70% (v/v) ethanol solution followed by disinfection with 1% (v/v) sodium hypochlorite (NaClO) and 0.1% (v/v) Tween-20 for 6 min. All the seeds were rinsed five times with sterile distilled water and germinated aseptically in Petri dishes containing germination medium consisting of semi-solid half-strength Murashige and Skoog medium (MS) [40] with 2% (w/v) sucrose and 0.7% (w/v) agar (pH 5.7). The seeds were maintained for 4 days at 4 °C in the dark and, afterwards, transferred to a growth chamber at 22 ± 2 °C or 15 ± 2 °C, both with a 16 h photoperiod, under cool, white fluorescent light (35 µmol m^−2^ s^−1^). The percentage of germination was recorded after 30 days.

#### 2.4.2. Shoot Multiplication

Seedlings were transferred to glass flasks containing germination medium, and when they reached approximately 100 mm in length, the shoots were removed, cut transversely into 2 sections, and each section was then transferred to MS medium [40] supplemented with 3% (w/v) sucrose and one of the following combinations of growth regulators: (i) 0.3 mg L^−1^ benzylaminopurine (BAP) plus 0.03 mg L^−1^ naphthaleneacetic acid (NAA); (ii) 0.2 mg L^−1^ BAP plus 0.02 mg L^−1^ NAA; (iii) 0.1 mg L^−1^ BAP plus 0.01 mg L^−1^ NAA. The medium was solidified with 0.7% (w/v) agar (pH 5.7), and the cultures were maintained in a growth chamber at 22 ± 2 °C and a 16 h photoperiod, with white fluorescent light (35 µmol m^−2^ s^−1^) and subcultured every 21 days. The multiplication rate was calculated at the end of each subculture (multiplication cycle) according to the formula (1):

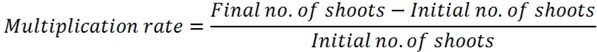
(1)

#### 2.4.3. Rooting and Acclimatization

For root induction, plants were transferred to MS medium [40] supplemented with 2% (w/v) sucrose and 0.7% (w/v) agar without growth regulators and maintained on rooting medium for at least 2 weeks. Rooting was expressed in terms of rooting frequency, root number and the longest root length per plantlet. *Rooted plantlets* were transferred into plastic pots containing either a mixture of soil, peat and perlite (1:1:1, v/v) or the commercial substrate, PINDSTRUP Universal. The 4-week-long acclimatization phase took place in a plant growth chamber maintained at 22 ± 2 °C and a 16-h light period with a light intensity of 35 µmol m^−2^ s^−1^. Several recipients containing water were placed inside the chamber to maintain high humidity, and potted plants were initially covered by plastic film and watered every day. Relative humidity was decreased by gradually opening the covers, which were completely removed after 2–3 weeks.

### 2.5. Statistical Analysis

Mean germination percentage comparisons were done with the Student’s *t*-test. Differences between the mean multiplication rate among different culture media was analyzed with a one-way ANOVA. Test assumptions, namely, the normality of data and the homogeneity of variances, were evaluated with the Shapiro-Wilk test and the Levene test, respectively. Statistical significance was assumed for *p* < 0.05.

The results reported in this work represent the average of at least three independent experiments and are represented as the mean ± SD. Differences among treatments were detected by analysis of variance with the Tukey HSD (Honestly Significant Difference) multiple comparison test (α = 0.05) for HPLC data, one way ANOVA for the phenolic content of hydroethanolic extracts of wild and *in vitro* propagated plants or a paired *t*-test for chemical characterization data between the first and second year. All statistical analyses were performed using SigmaStat 3.10 (Systat) software (Systat software Inc., Copyright^©^ 2004, Erkrath, Germany).

## 3. Results and Discussion

### 3.1. Chemical Characterization and Antioxidant Capacity of Wild *A. lopesianum*

For two consecutive years, the total phenolic content, total flavonoid content and the bioactivity antioxidant capacity were determined for the leaves and stems’ hydroethanolic extracts from wild *A*. *lopesianum* (Table 1). This is the first report of the chemical characterization of this species or any other *Antirrhinum* species. The assays described in this work are simple and rapid methods able to give an overview of the whole complex of substances possessing antioxidant properties in order to assess the potential of *A*. *lopesianum* leaves as a source for bioactive substances. In general, plant extracts present a positive relationship between total phenol content, flavonoid content and antioxidant capacity, with higher phenol and flavonoid levels reflecting greater antioxidant capacity. In addition, the comparison of these values among two consecutive years represents the added value of the analysis performed: significant differences were only denoted for the antioxidant capacity of the hydroethanolic extracts between the first and second year. A decrease in this factor may be a sign of environmental stress, which may have altered the plant metabolism [41,42,43]. Since we do not have differences in total phenol content, this decrease in antioxidant capacity may reflect other components contributing to this alteration, like minerals and/or organic acids.

**Table 1:** Chemical characterization of the hydroethanolic extracts of *A*. *lopesianum* in two consecutive years: **I**, after one year of growth at the greenhouse; and **II**, after two years of growth at the greenhouse. Total phenol content, expressed in milligrams of gallic acid equivalents (GAE) by gram of dry weight, flavonoid content, expressed in milligrams of catechin equivalents (CE) per gram of dry weight, and antioxidant capacity, expressed in nanomoles of Trolox equivalents (TE) per 100 g of dry weight, are presented. The values correspond to the average of at least three independent measurements ± standard deviation. Differences between the first and second year are denoted as ** *p <* 0.01.

Chemical Parameters	I	II
Total phenolic content (mg GAE g^−1^ dw)	5.545 ± 0.20	6.132 ± 0.56
Flavonoid content (mg CE g^−1^ dw)	0.673 ± 0.04	0.705 ± 0.04
Antioxidant capacity (nmol TE 100 g^−1^ dw)	29.115 ± 3.72	11.956 ± 1.16 **

### 3.2. Bioactivities Assessment

Various natural products are reported to have substantial neuroprotective activity, due to their radical scavenging capacity and AChE inhibitory activity. Both activities are important in the amelioration of neurodegeneration [5,6,8]. The undesirable side effects of compounds in pharmacological use make it important to identify natural neuroprotective molecules. AChE inhibition may help in the treatment of several neuropathologies, such Alzheimer’s disease (AD), as well as senile dementia, myasthenia gravis [13], Parkinson’s disease [14] and ataxia [15], due to the associated cholinergic deficit [12]. Few AChE inhibitors have yet been approved for AD therapy [12], and, therefore, natural sources of compounds exhibiting AChE inhibitory effects must be pursued.

In order to evaluate the AChE inhibitory activity of *A*. *lopesianum*, hydroethanolic extracts and a polyphenol-enriched fraction were evaluated, as described earlier [38]. This method allows the enrichment in polyphenols of the total extract, excluding the organic acids and sugars [35,44] The obtained results for AChE inhibition of *A*. *lopesianum* hydroethanolic extract (Table 2) are comparable to those described for other plants [15,38,39]. Other members of the *Plantaginaceae* family, to which *Antirrhinum* also belongs, have been shown to possess a significant anticholinesterase and antidementic properties, which may be useful in the treatment of dementia (*Bacopa monnieri* (L.) Pennell [45]; *Plantago major* subsp. *intermedia* [46]), validating the importance of studying AChE inhibitory activity in *A*. *lopesianum*. Well-known AChE inhibitors include alkaloids (physostigmine, galantamine), but other sources have been described, such as urosolic acid [47], lignans [48], flavonoids, terpenoids and coumarins [8]. The SPE fractionation performed on *A*. *lopesianum* hydroethanolic extract to obtain a polyphenol-enriched fraction presented inhibitory activity higher than the total extract, suggesting that polyphenols are the phytochemicals that can be associated with AChE inhibitory activity (Table 2).

**Table 2:** Acetylcholinesterase inhibitory activity of hydroethanolic extracts and the polyphenol-enriched fraction of *A*. *lopesianum*. Acetylcholinesterase (AChE) inhibition is presented as the percentage of inhibition using 1 and 2 mg mL^−1^ of extract. Values are the mean of three independent replicates ± standard deviation.

	Hydroethanolic extract	Polyphenol-enriched fraction
**% AChE inhibition** (2 mg mL^−1^)	37.48 ± 9.04	63.66 ± 7.11
**% AChE inhibition** (1 mg mL^−1^)	25.00 ± 9.70	32.57 ± 9.02

Based on this, *A*. *lopesianum* PEF was revealed to be a promising source of biomolecules with potential neuroprotective capacity, and therefore, it was tested for its intracellular antioxidant properties in a human neurodegeneration cell model.

**Figure 1:**
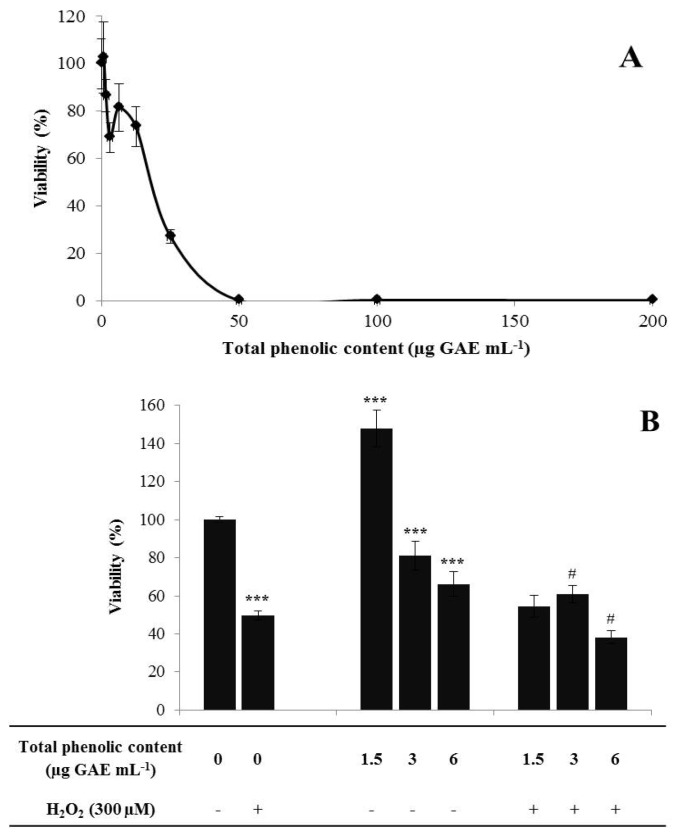
Cytotoxicity and neuroprotection of *A. lopesianum* sp. polyphenol-enriched fraction (PEF). (**A**) Cytotoxicity profile. Cell viability was determined for SK-N-MC neuroblastoma incubated with *A*. *lopesianum* sp. PEF (0–200 µg GAE mL^−1^) for 24 h. (**B**) Cytoprotection, cell viability expressed as a percentage of metabolic viable cells. Neuroblastoma cells were incubated with PEF for 24 h and then injured by 300 µM H_2_O_2_ for 24 h. Statistical differences compared with untreated cells are denoted as *** *p <* 0.001. Statistical differences compared with injured cells are denoted as ^#^ *p <* 0.05. All values are the mean ± SD, *n* = 6.

Toxic ranges of the extract were defined using a neuroblastoma cell line for a range of 0–200 µg GAE mL^−1^ for 24 h [39]. To cause a complete cell death, a concentration of 50 µg GAE mL^−1^ *A*. *lopesianum* PEF was required (Figure 1A). A concentration of approximately 20 µg GAE mL^−1^ *A*. *lopesianum* PEF was required to attain 50% cell viability.

After determining the range of nontoxic concentrations, three concentrations of *A*. *lopesianum* PEF were selected and tested in a neurodegeneration cell model to assess their cytoprotective effects. This model consists of neuroblastoma cells injured with 300 µM H_2_O_2_ for 24 h, a condition that routinely reduces cell viability by 50% [39]. Cell viability was assessed using the CellTiter-Blue^®^ Cell Viability Assay (Promega), a colorimetric method to determine cell metabolic activity.

The results show that under H_2_O_2_ stress, 3 µg GAE mL^−1^ of *A*. *lopesianum* PEF present a cytoprotective effect, significantly enhancing cell viability when compared with cells treated only with H_2_O_2_ (Figure 1B). On the other hand, the higher concentration of *A*. *lopesianum* PEF, 6 µg GAE mL^−1^, was no longer neuroprotective. Interestingly, when cells were incubated only with 1.5 µg GAE mL^−1^ of *A*. *lopesianum* PEF, cell viability increases relative to the control. This fact can be explained by the activation of a hormetic dose-response. Hormesis describes a process in which exposure to a low dose of an agent that is toxic at higher doses induces a beneficial effect on the cell, which is confirmed by an increase in cell viability [49]. These results are consistent with the results obtained for AChE inhibitory activity, suggesting that *A*. *lopesianum* PEF presents a neuroprotective effect. This is the first report for bioactivities related to the amelioration of the neurodegeneration process for *A*. *lopesianum* metabolites.

Moreover, the bioactive characterization reveals the potential of *A*. *lopesianum* for the production of bioactive compounds. Due to *A*. *lopesianum*’s endangered status, it is crucial to ensure its conservation and to study its bioactive production potential in propagated plants. *Ex situ* conservation and *in vitro* propagation methods were developed for the species.

### 3.3. *Ex situ* Conservation and *in vitro* Propagation

**Figure 2:**
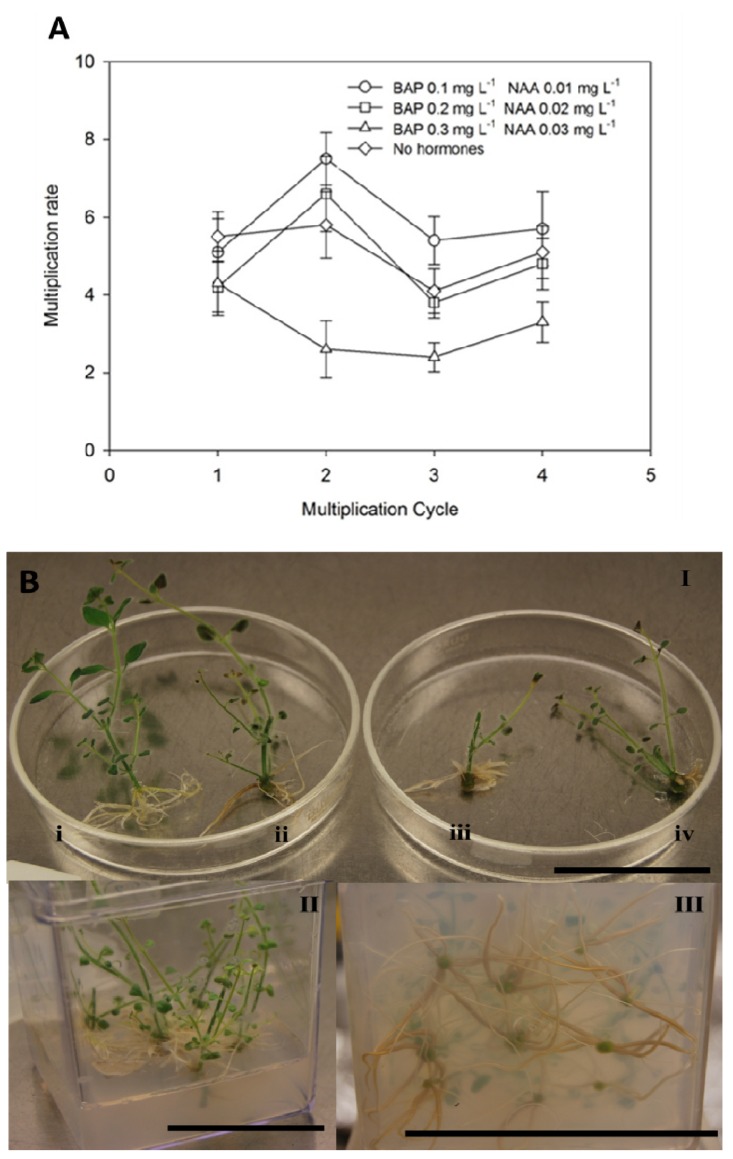
Multiplication rates and aspects obtained for *A*. *lopesianum* after each period of 21 days of culture (multiplication cycle). (**A**) Multiplication rates calculated according to formula (1) on media supplemented with the indicated different concentrations of benzylaminopurine (BAP) and naphthaleneacetic acid (NAA). Differences among multiplication cycles are only revealed to be statistically significance between the second and third multiplication cycles; differences among treatment reveal that only BAP 0.3 mg L^−1^ plus NAA 0.03 mg L^−1^ had a significant lower multiplication rate. (**B**) (I) *In vitro* plantlets obtained on medium without growth regulators (**i**), with BAP 0.1 mg L^−1^ plus 0.01 NAA mg L^−1^ (**ii**), with BAP 0.2 mg L^−1^ plus NAA 0.02 mg L^−1^ (**iii**) and with BAP 0.3 mg L^−1^ plus NAA 0.03 mg L^−1^ (**iv**). (II) Rooted shoots of *A*. *lopesianum* obtained after 21 days on medium without growth regulators. (III) Detail of induced roots. The bars correspond to 5 cm.

*A*. *lopesianum* collected seeds were sent to the Seed Bank A. L. Belo Correia, Museu Nacional de História Natural, and stored there, accordingly, with international standards for long-term seed conservation [50]. Seed germination was tested at 15 °C and 22 °C, the germination percentage being similar for both conditions (around 60%). After germination, shoots from *A*. *lopesianum* seedlings were cultured on MS solid medium supplemented with varying levels of BAP in combination with NAA for shoot multiplication. Regenerated shoots were evaluated at the end of each of the fourth multiplication cycles (21 days per cycle) (Figure 2). There were statistically significant differences in the mean multiplication rates both between treatments (*F*(3,144) = 10.939; *p <* 0.001), as well as multiplication cycles (*F*(3,144) = 3.869; *p* = 0.011). At the end of the first multiplication cycle, the multiplication rate was not significantly different among treatments, although a slightly higher multiplication was obtained on medium without growth regulators. The highest propagation was observed at the end of the second multiplication cycle with an average of 7.5 shoots obtained per initial shoot on medium with the lowest concentration of growth regulators. Tukey Post Hoc tests revealed that only the second and third multiplication cycles had statistically significant different mean multiplication rates. Afterwards, multiplication rates decreased and tended to stabilize at the end of the fourth multiplication cycle. By this time, while low concentrations of growth regulators or their omission showed the best results, the medium supplemented with BAP 0.3 mg L^−1^ plus NAA 0.03 mg L^−1^ was significantly less effective than the other treatments. In *A*. *majus*, it has been reported that the presence of cytokinins (kinetin or BAP) in the medium seems to be favorable for multiple shoot formation [51,52], but high concentrations of growth regulators used individually or in combination usually lead to the formation of callus with occasional abnormal shooting [53]. For *A*. *lopesianum*, the results obtained here also suggest that prolonged exposure to the higher concentrations of growth regulators tested might have a negative effect on shoots, leading to poor propagation ability. Similar observations have been previously reported for other species [54]. In this work, and considering that our main objective was to achieve efficient rooting in order to acclimatize the plantlets for *ex vitro* transfer and not the large-scale propagation of *A*. *lopesianum*, the absence of growth regulators might be advantageous. In fact, multiplication and rooting was successfully achieved in one step by culturing the shoots on medium devoid of growth regulators (Figure 2). One hundred percent of the shoots coming from any multiplication medium had developed several roots after 1–2 weeks on rooting medium. However, in the case of shoots exposed to growth regulators during multiplication, callus formation or browning of the basal part of the stem was observed. Furthermore, the number of roots per shoot, as well as the length of the longest root, was slightly smaller on the medium with a higher concentration of growth regulators, although these values were not significantly different among the shoots coming from different multiplication media. Each shoot formed on average of eight roots, with the length of the longest root being approximately 5 cm. During rooting, shoot elongation was more pronounced in shoots coming from multiplication medium without growth regulators (data not shown).

At the end of the rooting period, plantlets were transplanted into plastic pots containing a mixture of soil, peat and perlite (1:1:1, v/v) in order to acclimatize them to field conditions. Acclimatization was successful, with 90% of the plantlets surviving this process and presenting constant growth during the four weeks of acclimatization. At the end of this period, live specimens were sent to several Portuguese botanic gardens (Jardim Botânico da Ajuda, Jardim Botânico do Museu Nacional de História Natural, Jardim Botânico de Coimbra, Jardim Botânico da Universidade de Trás-os-Montes e Alto Douro), and some specimens were transported and re-introduced into the natural habitat in the Bragança region.

#### Chemical Characterization and Antioxidant Capacity of *in vitro* Propagated Plants

**Figure 3:**
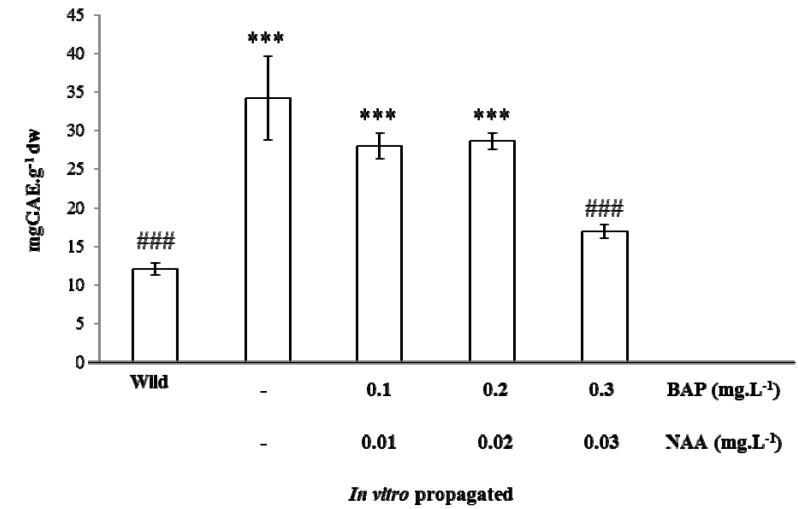
Total phenolic content of hydroethanolic extracts of wild and *in vitro* propagated plants of *A*. *lopesianum*, with and without growth regulators. The values are expressed in milligrams of GAE per gram of dry weight and correspond to the average of at least three independent measurements ± SD. Differences between treatments in relation to wild plants are denoted as *** *p <* 0.001. Differences between treatments in relation to *in vitro* propagated plants without growth regulators are denoted as ^###^ *p <* 0.001.

A comparison between the total phenolic content of wild and *in vitro* propagated plants was also performed in order to evaluate the ability of *A*. *lopesianum* to keep the capacity of phytochemical production *in vitro*, with and without growth regulators, relative to the second year data (Figure 3). Interestingly, the *in vitro* propagated plants presented a higher content in total phenols, but increasing concentrations of growth regulators led to a decrease in the total phenol content. This is in accordance with the trend observed for the multiplication rates in the same conditions, where higher amounts of BAP and NAA led to a lower multiplication rate. Therefore, our data suggest that the production ofsecondary metabolites by *in vitro* plants is favored under conditions that promote active propagation. Alternatively, it may be possible that the presence of growth regulators in the culture medium negatively affects secondary metabolite production.

Our data is in agreement with other works in which it was described that *in vitro* propagated plantlets of snow lotus (*Saussurea*) presented much higher polyphenol content than their wild counterparts [55], suggesting important possible future applications of *in vitro* propagated plants relatively to wild ones for bioactives production. Furthermore, Danova and co-workers [56] reported that growth regulator supplementation in *Hypericum rumeliacum in vitro* culture led to a decrease of total phenolics and flavonoid content in comparison with growth regulator-free medium. Since the *in vitro* propagation of *A*. *lopesianum* in the absence of growth regulators seems to be the most promising approach for future studies, the chemical characterization of this species was refined by HPLC profiling.

**Figure 4:**
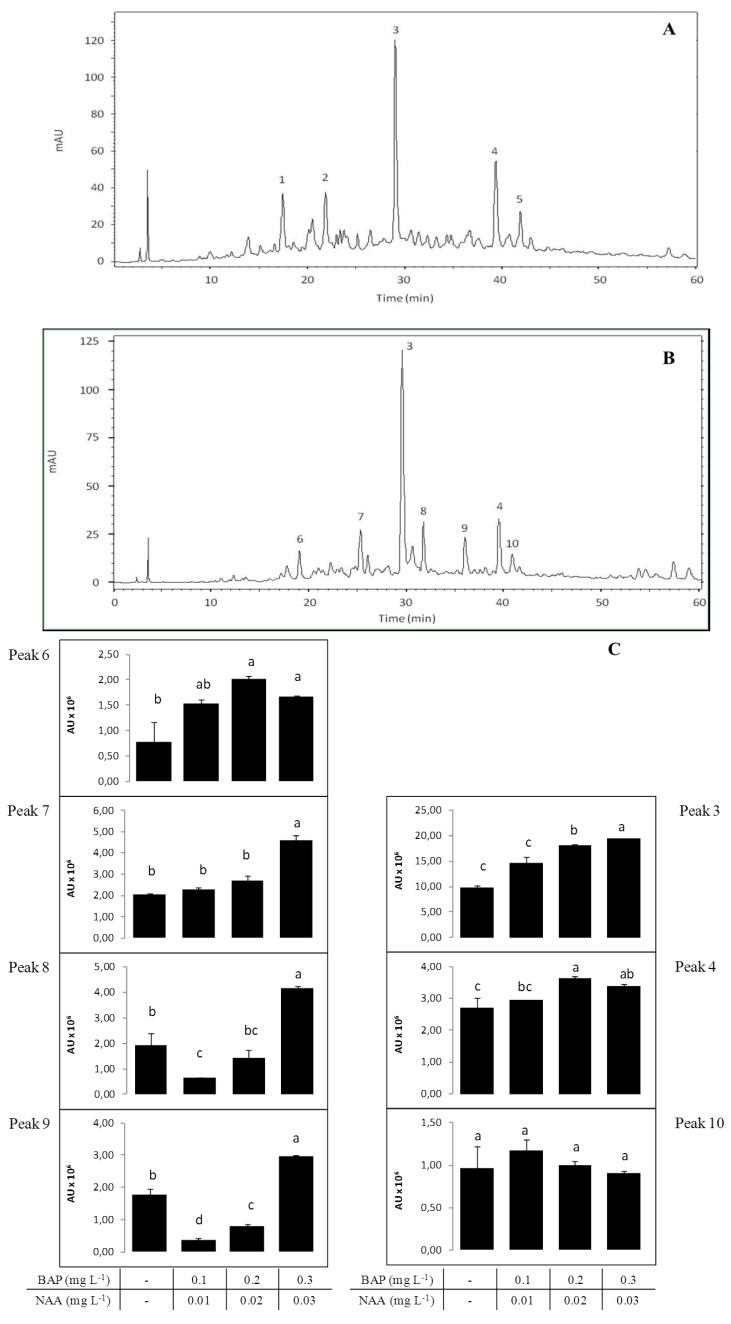
HPLC spectrum at 280 nm of wild (**A**) and micropropagated plants without growth regulators (**B**); the numbers correspond to identified compounds, namely, cinnamic acid derivatives (1, 2 and 5), flavones (3, 4 and 10) and simple phenolic acids derivatives (6, 7, 8 and 9). Units are expressed as arbitrary absorbance units (mAU). (**C**) Compound levels, derived from simple phenolic acids (6, 7, 8 and 9) and flavones (3, 4 and 10) identified by HPLC at 280 nm for *A*. *lopesianum* with and without growth regulators in different concentrations. Units are expressed as millions of arbitrary absorbance units (AU × 10^6^). Values are the average of at least three independent replicates.

The HPLC profiles, of leaves and stems from wild and micropropagated plants of *A*. *lopesianum*, were compared. Tentative identification of the class of the compounds by HPLC (Figure 4) has been made by examination of their UV spectra. Each class of flavonoids has a typical UV absorption maxima, and the classification proposed by Robards and Antolovich has been used in this work for identification purposes [57]. Flavones typically exhibit an intense band II (310–350 nm) absorption maximum with a shoulder or low intensity peak representing band I (250–280 nm) [58]. Flavonols absorb at 250–280 nm (band II) and 350–385 nm (band I), while hydroxycinnamic acids lack band I and exhibit absorption at 227–245 nm and at 310–332 (band II).

The HPLC analysis showed quantitative and qualitative differences in the phytochemicals for wild and micropropagated plants of A. *lopesianum* (Figure 4A,B). From the five principal peaks identified in wild plants (Figure 4A), only flavones (peaks 3 and 4) are kept in micropropagated plants. Plants micropropagated without growth regulators present seven major peaks (Figure 4B), the most identified being derived from simple phenolic acids (6, 7, 8 and 9) and the remaining, flavones (3, 4 and 10). We also detected that the conditions used for the *in vitro* propagation had an effect on these major produced phytochemicals (Figure 4C). Interestingly, we observe a differential effect of the plant growth regulators in areas of the mentioned peaks that is more pronounced for phenolic acid derivatives. For instance, for phenolic acid 6, there is an increase upon the addition of different growth regulator combinations relative to the control (without growth regulators). On the other hand, for phenolic acid 7, only the addition of BAP 0.3 mg L^−1^ plus 0.03 NAA mg L^−1^ generated a statistically significant increase. Phenolic acids 8 and 9 present a reduction of the concentrations of BAP 0.1 mg L^−1^ plus 0.01 NAA mg L^−1^ and BAP 0.2 mg L^−1^ plus 0.02 NAA mg L^−1^ and an increase for the higher concentration of growth regulators. On the other hand, the effect of growth regulators on the identified flavones was not so pronounced, and the amounts hardly varied under the different concentrations of growth regulators.

## 4. Conclusions

Chemical characterization and detection of bioactive compounds of *A*. *lopesianum* reveal a hidden potential of this endemic species that has not yet been properly valued. *A*. *lopesianum* presents potential as a source of natural compounds with AChE inhibitory and neuroprotective activities.Therefore, *A*. *lopesianum* extracts deserve to be the target of bioguided fractionation, and further characterization must be conducted, because those bioactivities contribute to the value of the specie as a natural source of neuroprotective compounds.

Moreover, we found that the best conditions for efficient propagation of *A*. *lopesianum*, lacking growth regulators, are simultaneously the ones leading to total higher contents of phenolic compounds. Nevertheless, differential accumulation of phenolic acid derivatives was detected in the presence of growth regulators, a not so obvious effect for flavones. This opens new avenues for the plant production of phytochemicals, since limited data is available on the impact of growth regulators on secondary metabolite production *in vitro*. Differential accumulation of specific secondary metabolites can also be explored through the manipulation of growth regulators in the culture medium and, possibly, other types of medium components, in order to increase the amounts of specific compounds that may reveal interest in potential applications.
